# Saikosaponin-d increases radiation-induced apoptosis of hepatoma cells by promoting autophagy via inhibiting mTOR phosphorylation

**DOI:** 10.7150/ijms.53024

**Published:** 2021-01-29

**Authors:** Baofeng Wang, Weili Min, Shuai Lin, Lingqin Song, Pengtao Yang, Qingyong Ma, Jian Guo

**Affiliations:** 1 Department of Radiation Therapy, Second Affiliated Hospital, Xi'an Jiaotong University, Xi'an 710004, China.; 2 Department of Surgical Oncology, Second Affiliated Hospital, Xi'an Jiaotong University, Xi'an 710004, China.; 3 Department of Hepatobiliary Surgery, First Affiliated Hospital, Xi'an Jiaotong University, Xi'an 710061, China.

**Keywords:** Saikosaponin-d, Radiation, Autophagy, mTOR.

## Abstract

**Objective:** The aim of this study was to analyze the effects of saikosaponin-d (SSd) on autophagy activity and radiosensitivity of hepatoma cells, and to elucidate its related molecular mechanisms.

**Methods:** The growth of SMMC-7721 and MHCC97L hepatoma cells were detected by clonal formation and survival fraction. Flow cytometry was used to detect the changes of apoptosis of hepatoma cells. The morphological changes of autophagy of hepatoma cells were observed by transmission electron microscopy and were further quantitatively detected by laser confocal microscopy. The expressions of related proteins were detected by Western blotting.

**Results:** SSd can significantly increase the apoptosis of hepatoma cells induced by radiation and inhibit the proliferation of hepatoma cells. The addition of the autophagy inhibitor chloroquine (CQ) or an mTOR agonist (MHY1485), which could reduce the promoting effect of SSd on radiation-induced apoptosis and inhibitory effect on the proliferation of hepatoma cells. Transmission electron microscopy and confocal microscopy results also showed that the number of autophagosomes was significantly higher in the radiation and SSd co-treatment group than in the radiotherapy or SSd alone group; however, the effect of SSd on autophagy in hepatoma cells was decreased after adding MHY1485, siRNA-P53 or AMPK inhibitor (Compound C). Western blot analysis showed that after the addition of SSd, the phosphorylation of mTOR was significantly decreased by radiation, the expression of the autophagy-related proteins LC3-II and Beclin-1 was increased, p62 was decreased, and the expression of cleaved caspase-3 and cleaved PARP was enhanced; this effect of SSd was partially reversed after the addition of MHY1485, siRNA-P53 or Compound C.

**Conclusions:** SSd increases radiation-induced apoptosis of hepatoma cells by promoting autophagy via inhibiting mTOR phosphorylation and providing a possible potential approach for radiosensitization therapy of liver cancer.

## Introduction

Primary liver cancer is one of the most common malignant tumors in the world. Approximately 90% of liver cancer is hepatocellular carcinoma, ranking fifth among malignant tumors worldwide, and its mortality ranks second among all cancers 1. The incidence and mortality of liver cancer increase annually. Eastern Asia and Southeast Asia, particularly China, are the areas with the highest incidences of liver cancer 2. Currently, liver cancer treatment is still surgery-based, but patients with middle and late stage disease have often lost the opportunity for operation and can choose only other non-surgical treatment methods. Non-surgical treatment of liver cancer includes mainly transcatheter arterial chemoembolization (TACE), radiation therapy, radiofrequency ablation, targeted therapy and immunotherapy 3, but their curative effects are limited. The 5-year survival rate for liver cancer is only approximately 5%. Most patients die within 1 year after diagnosis 4. In recent years, radiotherapy has proven to be a safe and effective treatment for liver cancer, especially stereotactic radiotherapy, which has resulted in great progress in the treatment of liver cancer 5, 6. Recent studies have found that radiotherapy for liver cancer can cause changes in the level of autophagy, and effective regulation of autophagy can increase the radiosensitivity of liver cancer cells 7, 8. Therefore, further study of the role of autophagy in the treatment of liver cancer is of great significance.

Autophagy is a highly conserved process of self-digestion and cell survival that plays an important regulatory role in various physiological and pathological processes. Autophagy plays a dual role in promoting or inhibiting tumor growth at different stages of tumorigenesis. Studies have shown that when autophagy is absent or abnormal, the incidence of tumors can be greatly increased. After tumorigenesis, autophagy can promote the resistance of tumor cells to starvation and hypoxia and promote the survival of tumor cells in adverse environments 9,10. In other studies, it has been confirmed that increasing autophagy in tumor cells to a certain extent enhances the efficacy of radiation therapy 11, 12. The rapamycin target protein (mTOR) is a key target molecule involved in the regulation of autophagy function. mTOR inhibits autophagy by inhibiting ULK1 inactivation via phosphorylating serine at position 757 in ULK1. mTOR is regulated mainly by two upstream negative regulators, the AMPK and p53 signaling pathways. Under deficient cell nutrition conditions, the AMPK and p53 proteins can be activated, which further promote autophagy by inhibiting mTOR. Conversely, when cells are well nourished, intracellular AMPK and p53 expression is inhibited, which inhibits autophagy by activating mTOR 13, 14.

Saikosaponin is an active substance extracted from the Chinese herbal medicine Bupleurum. Saikosaponin has 9 types: a, b, c, d, e, f, g, h, and I. Saikosaponin-d (SSd) is one of the highest pharmacological activities 15. Previous studies have shown that SSd can inhibit tumor cell proliferation, promote tumor cell apoptosis, and increase liver cancer cell radiosensitivity 16, 17. In addition, SSd has been shown to have potential anti-cancer effects because it participates in the regulation of autophagic apoptosis in tumor cells 18.

Based on our previous experimental results, we used cell colony formation experiment and flow cytometry assays to observe the inhibitory effect of SSd and radiation on hepatoma cell growth and apoptosis induction. The effects of SSd and radiation on the autophagy of hepatoma cells were observed by transmission electron microscopy and laser confocal microscope. At the same time, western blotting was used to detect related protein expression changes. This study aims to investigate whether SSd mediates the radiosensitivity of hepatoma cells by regulating the level of autophagy, and provides experimental evidence for the radiosensitization research of liver cancer.

## Materials and Methods

### Cells and Reagents

Saikosaponin-d (≥98% purity) was purchased from Bencao Tiangong Technology Co., Ltd (Jiangxi, China). SMMC-7721 and MHCC97L human HCC cell lines were provided from the Medical Experimental Animal Center of the Fourth Military Medical University (Xi'an, China); Specific LC3, mTOR, p-mTOR, cleaved caspase-3, cleaved PARP, Beclin-1 and p62 monoclonal antibodies were purchased from Abcam (Cambridge, MA, United States). SiRNA fragment targeting p53 was from Ruibo Biotechnology Company (Guangzhou, China), Chloroquine (CQ), Compound C and MHY1485 were obtained from Sigma Chemical Co. (St. Louis, MO, USA). p53 polyclonal antibody was purchased from Cell SignalingTechnology Co. Ltd. (Boston, USA), Annexin V and PI Apoptosis Detection Kit was purchased from Yuheng Biotechnology Co., Ltd(Suzhou, China). The mRFP-GFP-LC3 fluorescent autophagy indicator system was purchased from Han heng Biotechnology Co., Ltd (Shanghai, China).

### Cell culture and intervention conditions

Cells were cultured in RPMI-1640 medium (PAA Laboratories GmbH, Austria) and supplemented with 10% fetal bovine serum (FBS) in a humidified atmosphere containing 5%CO_2_ at 37°C.Then, the cells were treated with radiation alone, SSd alone, or a combination of radiation and SSd. Radiation was performed at different doses (6 MV, and a dose rate of 400 cGy/min) by using an X-ray linear accelerator. SSd was also administered at concentrations (3µg/ml) as described previously 16, and added to the cultures at 2h before radiation. Chloroquine (CQ) (25μmol/L), mTOR inhibitor (MHY1485, 10μmol/L) or AMPK inhibitor (Compound C, 5µmol/L) was added 4 hours prior to radiation or SSd intervention. 24h after transfection of siRNA-P53 into liver cancer cells, the cells were routinely digested and collected to verify the expression of p53 gene. Control cultures received a carrier solvent consisting of 0.1% DMSO.

### Cell survival analysis

Cell colony formation experimental procedure refers to the previous method 19.The clonal formation rate = number of colonies in the intervention group / number of control colonies×100%. According to different irradiation dose gradients, different numbers of cells were inoculated into 6-well plates for intervention. Fit the cell survival curve according to the equation Y=1-(1-exp(-k*x))^N. Calculate the average cell lethal dose (D0), quasi-domain dose (Dq), 2Gy irradiated cell survival fraction (SF2), extrapolated number (N) and radiosensitization ratio (SER).K is a constant related to the quality of radiation and cell sensitivity. X is the radiation dose received by the cell. Y is the cell survival fraction. N is the target number, which reflects the number of radiation-sensitive regions in the cell. D0=1/k, SER=D0 without sensitizer group/D0 with sensitizer group. Plating efficiency (PE) = (number of clones in control group/number of cells inoculated)×100%. Surviving fraction(SF)= number of clones in experimental group /(number of cells inoculated×PE).

### Apoptosis assay

At the end of the intervention, the cell culture medium was discarded, and the cells were digested with trypsin to create a single cell suspension and counted in a cell counting plate. Subsequently, the binding buffer from the kit was added, and the cells were resuspended. Then, 5 μl of Annexin V-FITC (concentration: 20 μg/ml) and PI (concentration: 50 μg/ml) were added, and the cells were incubated at 4 °C in the dark for 30 min. The apoptosis rate of each group of cells was detected by flow cytometry (selection wavelength: 485/525 nm).

### Transmission electron microscopy

MHCC97L cells of each experimental group were washed twice with PBS. Cells were fixed in 100 mM phosphate buffer, pH 7.4, containing 25 g/L glutaraldehyde for 30 min at 4 °C. The cells were rinsed twice with ice-cold PBS, post-fixed in OsO4, dehydrated in graded acetone and embedded in epoxy resin. After slicing by the ultra-microtome, cells were stained by uranyl acetate and lead citrate. Photographs were obtained using transmission electron microscope.

### Western blot analysis

Western blot experimental procedure refers to the previous method 16.The protein concentration was detected using a BCA method. The blots were probed with antibodies against LC3 (diluted 1:2000), mTOR (diluted 1:800), p-mTOR (diluted 1:2000), cleaved caspase-3 (diluted 1:1000), AMPK (diluted 1:800), p-AMPK (diluted 1:800), cleaved PARP (diluted 1:1000), p53 (diluted 1:800), Beclin-1 (diluted 1:1000) and P62 (diluted 1:1000). The membranes were incubated in the dark with ECL (Amersham) to detect chemiluminescence. After blocking with 5% skim milk for overnight at 4 °C, the membrane was incubated for overnight with primary antibodies at 4 °C and then incubated with horseradish peroxidase-conjugated secondary antibodies (Nichirei Corporation, Tokyo, Japan). FX5 Spectra Imaging System was used to obtained blot images directly from PVDF membrane, and the optical density of the bands was measured via Image J software (NIH, Bethesda, MD, USA).The membranes were re-probed for β-actin as loading control.

### mRFP-GFP-LC3 fluorescence method for determining autophagy

MHCC97L cells were seeded in a 35-mm laser confocal culture dish and mRFP-GFP- LC3 adenovirus was infected according to the manufacturer's instructions. GFP is an acid-sensitive GFP protein, and mRFP is a stable fluorescent expression group that is not affected by external stimuli. A puncta is equivalent to an autophagosome, and the number of puncta can be counted to evaluate the level of autophagy activity.

### Statistical analysis

Statistical analysis was performed using SPSS 18.0 software. Graphpad Prism 5.0 software was used to fit the cell survival curve. Quantitative data were presented as the mean±SD. One-way analysis of variance (ANOVA) was used to compare data from different groups. Data were considered significant if *P*< 0.05.

## Results

### SSd enhances the inhibitory effects of radiation on hepatoma cell growth

**Figure 1:**
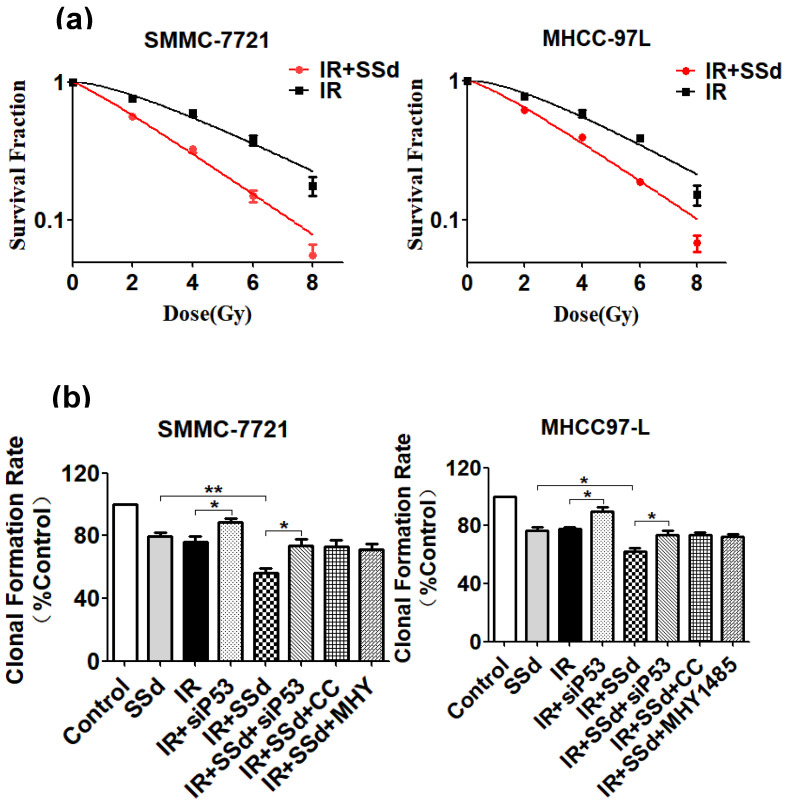
Changes in clonal formation rate of SMMC-7721 and MHCC-97L hepatoma cells after different interventions. **(a)** Effect of different interventions on survival curve of hepatoma cells; **(b)** Effects of different interventions on clonal formation rate of hepatoma cells. SSd: saikosaponin-d; IR: radiation; siP53: siRNA targeting p53 (siRNA-P53); CC: AMPK inhibitor (Compound C); MHY: mTOR agonist (MHY1485). Data are shown as mean±SD, *P< 0.05, **P< 0.01.

As our previous experimental results 19, after adding SSd, it can significantly enhance the inhibitory effect of radiation on the formation of hepatoma cell clones. To further confirm that SSd can increase the radiosensitivity of liver cancer cells, we fit the cell survival curve by clicking the multi-target mathematical model, and the results are shown in Figure 1. In the radiation alone group, SMMC-7721 hepatoma cells K=0.25, N=1.73, D0=4.0, Dq=2.92, SF2=0.76, and the combination of radiation and 3μg/ml SSd group K=0.34, N=1.22, D0=2.94, Dq=0.65, SF2=0.56, SER=1.36; Similarly, in the radiation alone group, MHCC-97L hepatoma cells K=0.26, N=1.88, D0=3.85, Dq=3.38, SF2=0.78. In the combined group K=0.32, N =1.37, D0=3.13, Dq=1.16, SF2=0.62, SER=1.23. The cell fitting survival curve further confirmed that the addition of SSd can significantly increase the sensitivity of liver cancer cells to radiation. After adding siRNA-P53, Compound C or MHY1485, the colony formation rates of SMMC-7721 cells were 73.7%, 72.8%, 71.2%, and the colony formation rates of MHCC-97L cells were 73.5%, 73.6%, and 72.2%, respectively. Significantly higher than the combined group (*P*<0.05, Figure 1). This indicates that siRNA-P53, Compound C and MHY1485 can significantly reverse the inhibitory effect of SSd on the formation of hepatoma cell clones under radiation conditions.

### SSd promotes radiation-induced apoptosis in liver cancer cells

**Figure 2:**
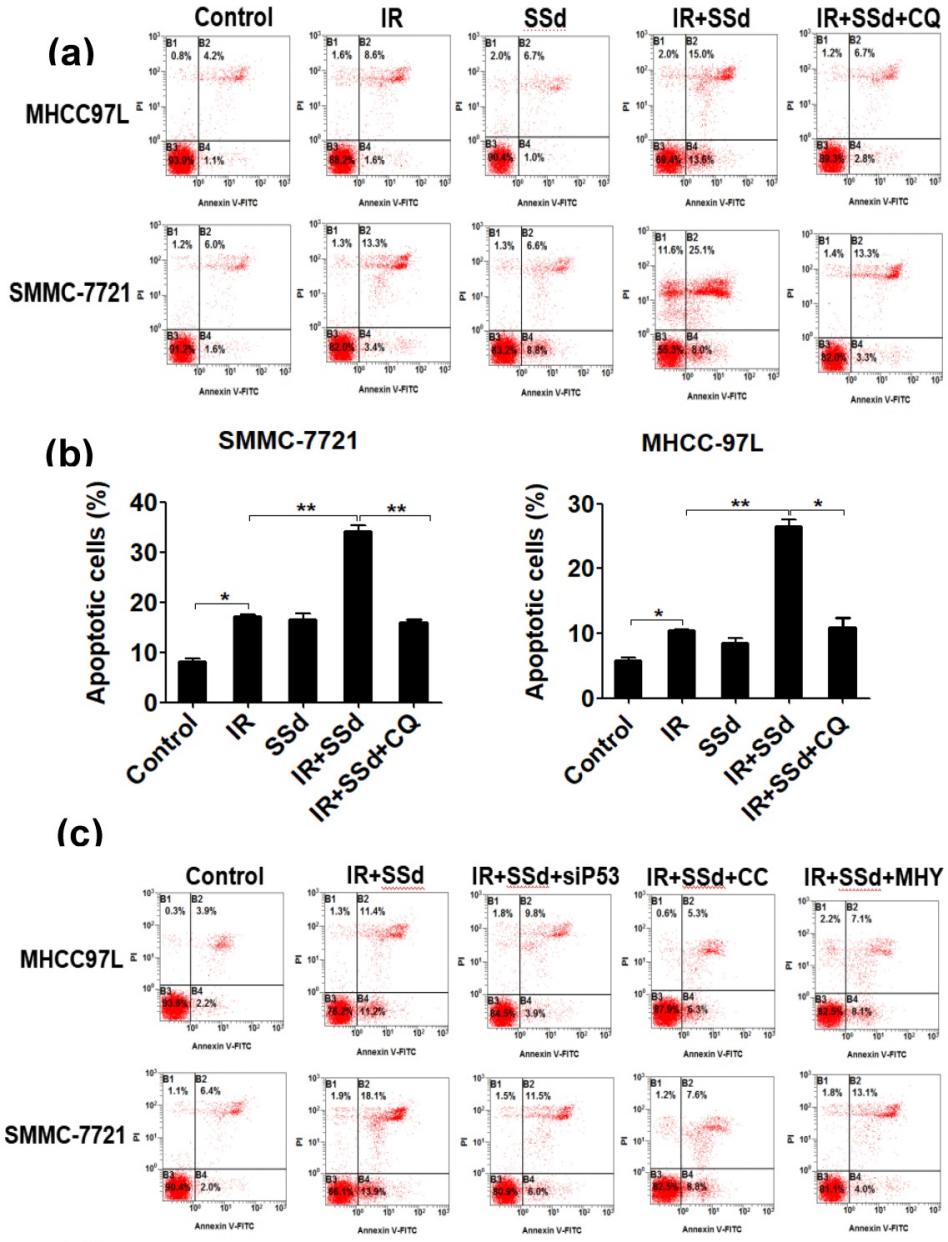

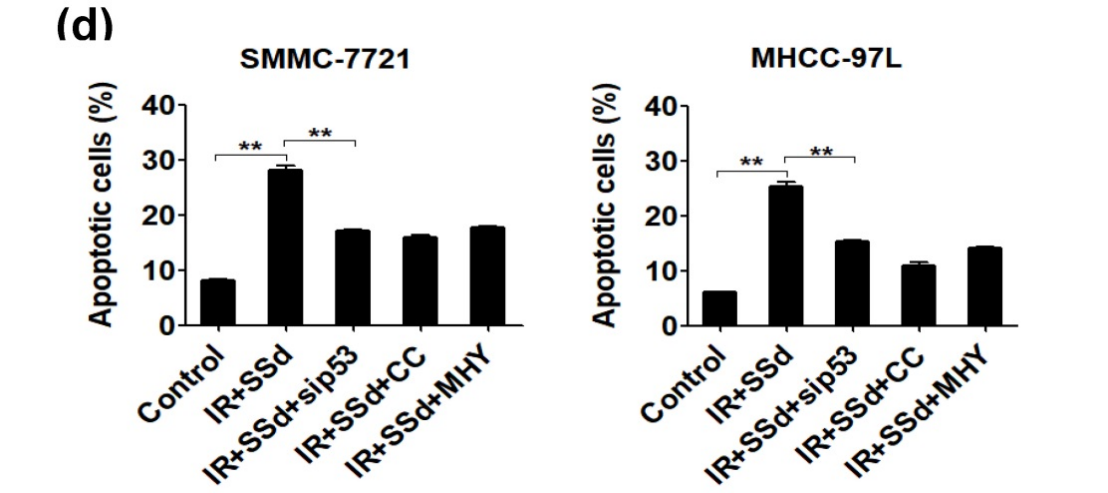
Effects of different interventions on apoptosis of SMMC-7721 and MHCC-97L hepatoma cells. **(a)** Flow cytometry shows apoptotic changes of hepatoma cells after different interventions; **(b)** Quantitative assessment of apoptotic ratio of hepatoma cells after different interventions; **(c)** Flow cytometry shows apoptotic changes of hepatoma cells after interference with mTOR; **(d)** Quantitative assessment of apoptotic ratio of hepatoma cells after interference with mTOR. SSd: saikosaponin-d; CQ:Chloroquine; IR: radiation; siP53: siRNA targeting p53 (siRNA-P53); CC: AMPK inhibitor (Compound C); MHY: mTOR agonist (MHY1485). Data are shown as mean±SD, *P< 0.05, **P < 0.01.

Flow cytometry results showed that the apoptosis rates for SMMC-7721 cells and MHCC-97L cells after radiation exposure were significantly higher than those of the control group (*P*<0.05). When SSd was added before radiation, the apoptosis of liver cancer cells was further increased (apoptosis rates of SMMC-7721 cells and MHCC-97L cells in the combined group were 34.20±1.10 and 26.45±1.15, respectively; *P*<0.01 vs. control group), indicating that SSd can enhance radiation-induced apoptosis in liver cancer cells. Subsequently, we added the autophagy inhibitor chloroquine, siRNA- P53, MHY1485 or Compound C to SSd and radiotherapy co-treatment. The effect of SSd on the radiation-induced apoptosis of hepatoma cells is significantly reduced. Compared with the combined group of radiation and SSd, there is a significant statistical difference (*P*<0.01, Figure 2). It further shows that inhibiting or interfering with mTOR-related molecules can reverse the promotion effect of SSd on radiation-induced apoptosis of liver cancer cells.

### SSd increases radiation-induced autophagosome formation in hepatoma cells

**Figure 3:**
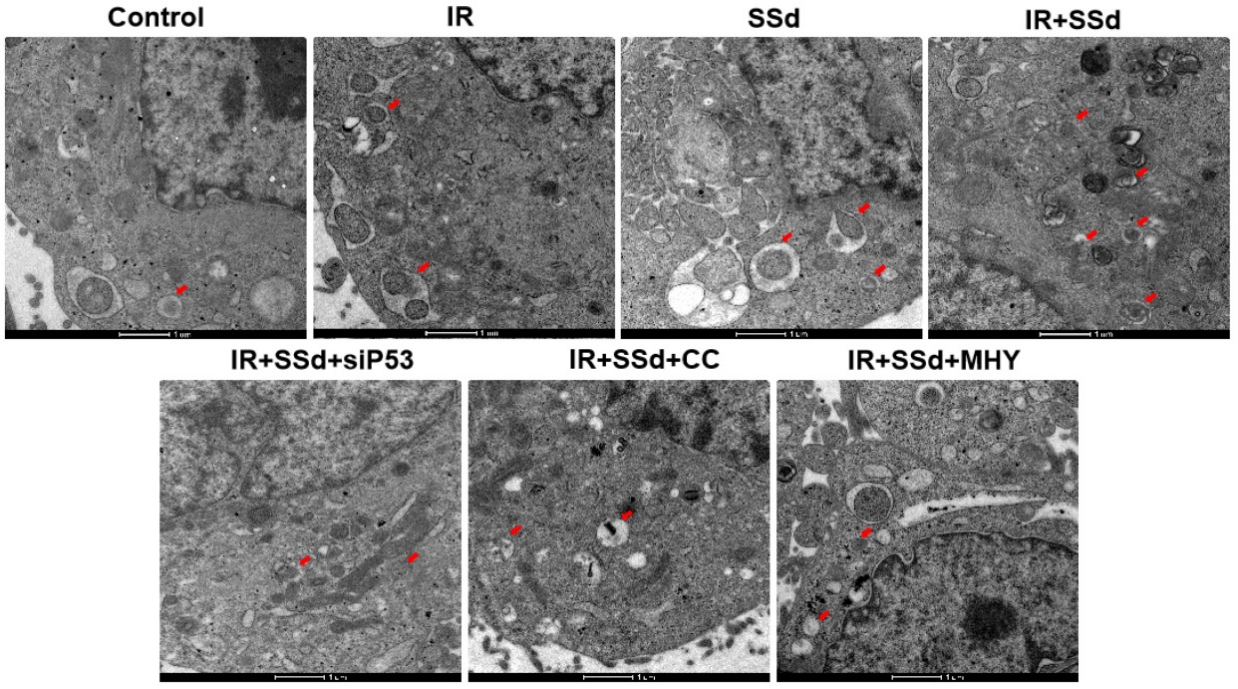
SSd increases radiation-induced autophagosome formation in MHCC-97L cells.Compared with the control group, the number of autophagosomes in MHCC-97L cells increased significantly after radiation exposure. After the addition of SSd, more autophagosomes in hepatoma cells were observed under transmission electron microscopy, and after the addition of the mTOR agonist MHY or AMPK inhibitor Compound C or siRNA-P53, the number of autophagosomes in the hepatoma cells was reduced in the combined group. SSd: saikosaponin-d; IR: radiation; siP53: siRNA targeting p53 (siRNA-P53); CC: AMPK inhibitor (Compound C); MHY: mTOR agonist (MHY1485). The scale bar represents 1μm.

As showed in Figure 3, when compared with the control group, the number of autophagosomes in MHCC-97L cells increased significantly after radiation exposure. When SSd was added, more autophagosomes in hepatoma cells were observed under transmission electron microscopy, indicating that SSd can enhance autophagy induced by radiation in liver cancer cells. Similarly, after the addition of the siRNA-P53, MHY1485 or Compound C, the number of autophagosomes in the hepatoma cells was reduced in the combined treatment group.

### The activation of mTOR abolished the enhanced effect of SSd on radiation-induced autophagosomes

**Figure 4:**
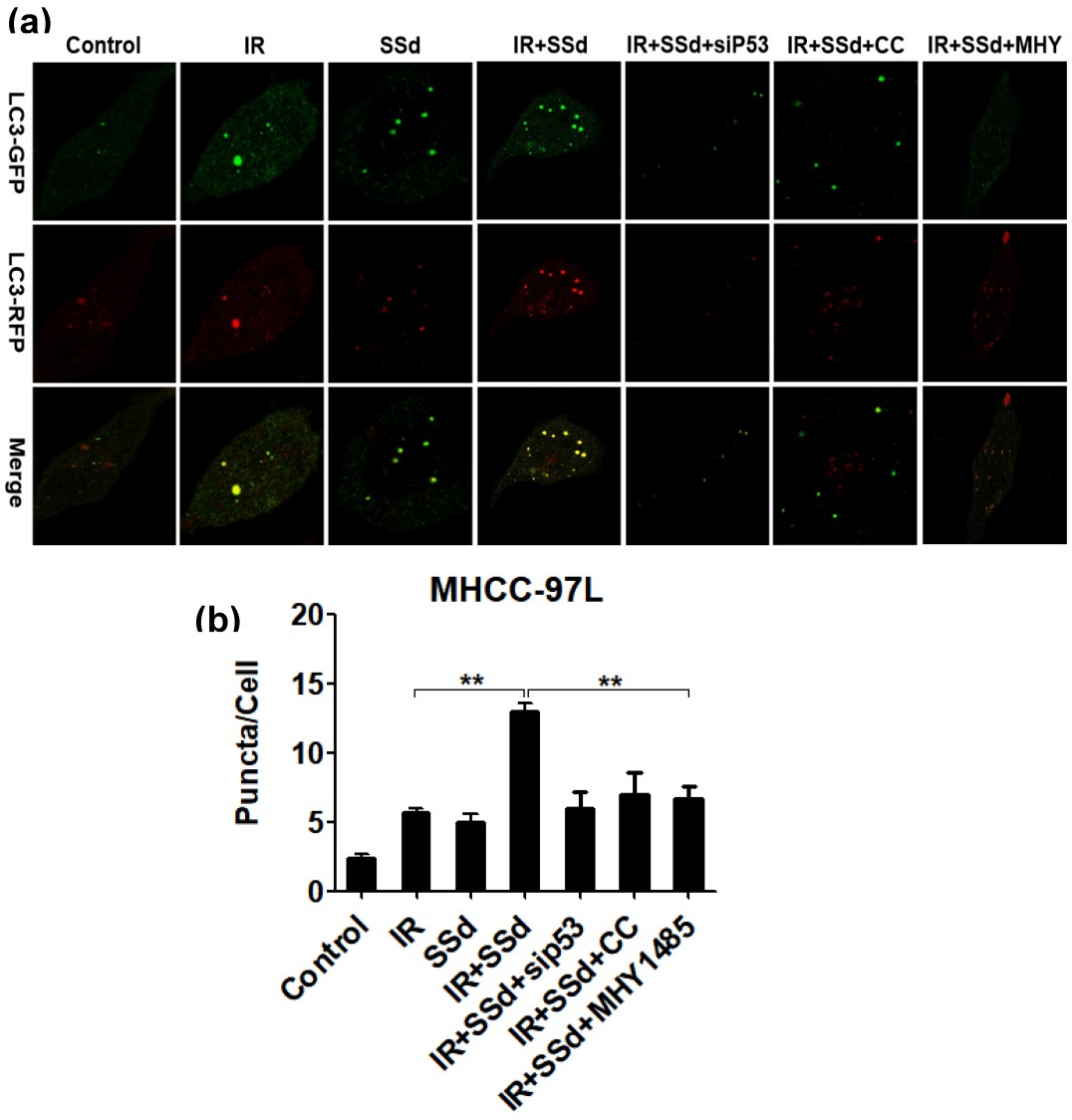
Laser confocal microscopy shows that SSd increases radiation-induced autophagosome formation in MHCC-97L cells. Representative photographs obtained from each group (a) and statistical analysis from all cells in each group (b).SSd can enhance radiation-induced autophagy formation on liver cancer cells. Similarly, after addition of siRNA-P53 or CC or MHY, the number of autophagosomes in MHCC-97L cells was decreased in the combined group. SSd: saikosaponin-d; IR: radiation; siP53: siRNA targeting p53 (siRNA-P53); CC: AMPK inhibitor (Compound C); MHY: mTOR agonist (MHY1485). Data are shown as mean±SD, **P < 0.01.

Based on transmission electron microscopy results, we also observed the formation of autophagosomes when hepatocarcinoma cells were transfected with mRFP-GFP-LC3 adenovirus. These results are shown in Figure 4. Compared with that in the control group, the number of autophagosomes in MHCC-97L cells increased significantly after treatment with radiation or SSd alone (*P*< 0.05), indicating that both radiation and SSd treatment can induce autophagy in MHCC-97L cells. However, when SSd was added prior to radiation, the number of autophagosomes in MHCC-97L cells was further increased. These results indicated that SSd can enhance radiation-induced autophagy formation in liver cancer cells (*P*<0.01). Similarly, after the addition of siRNA-P53, MHY1485 or Compound C, the number of autophagosomes in MHCC-97L cells was decreased in the combination treatment group (*P*<0.01). These results suggest that the addition of siRNA-P53, MHY1485 or Compound C can reverse the promotion of radiation-induced autophagy by SSd in hepatoma cells.

### The enhanced effect of SSd on radiation-induced hepatoma cell apoptosis may be related to promoting autophagy

**Figure 5:**
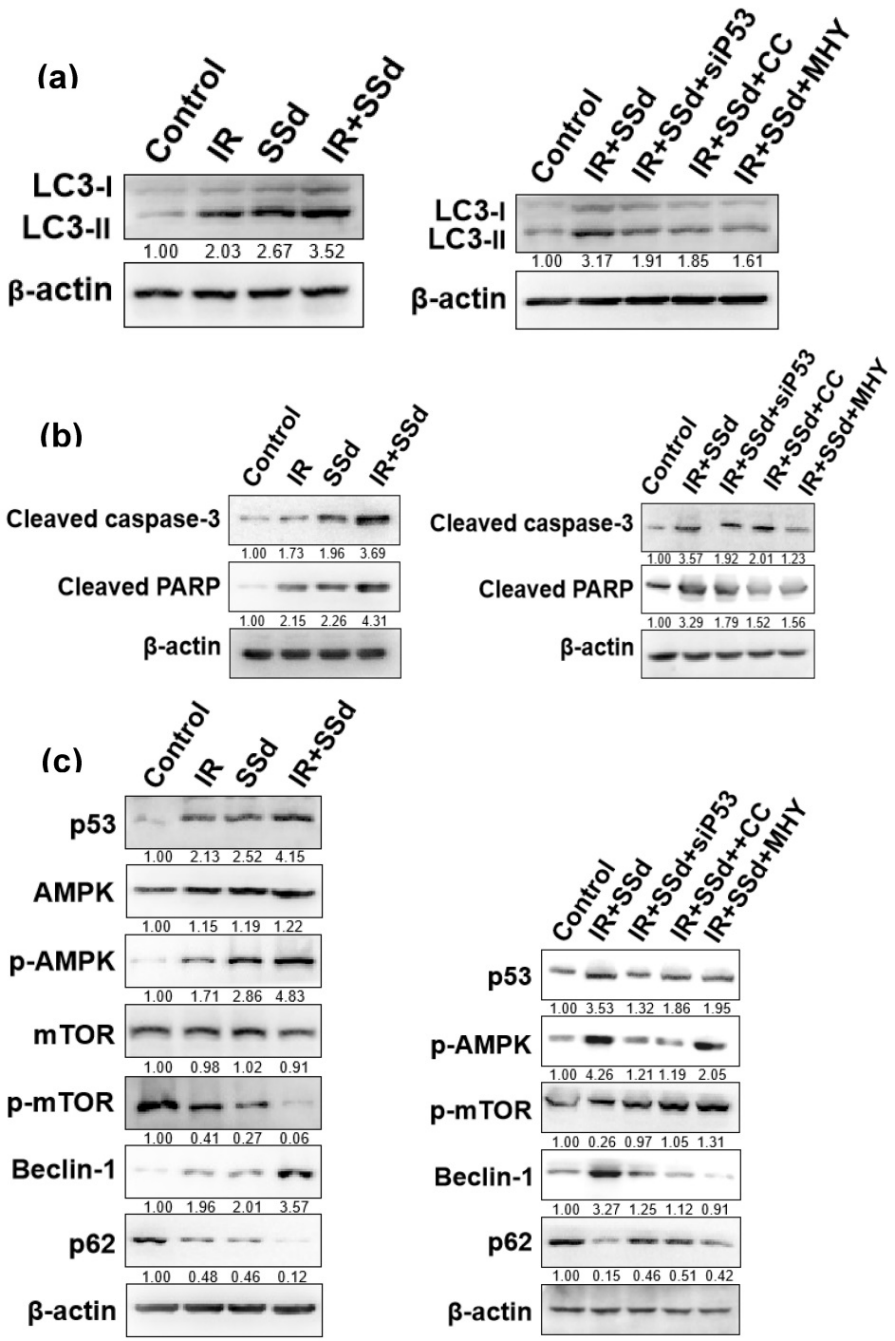
Effects of different interventions on related protein expression in MHCC-97L cells. **(a)** Changes of LC3-II expression after different interventions; **(b)** Changes of apoptosis-related protein expression after different interventions; **(c)** Changes of autophagy related protein expression after different interventions. SSd: saikosaponin-d; IR: radiation; siP53: siRNA targeting p53 (siRNA-P53); CC: AMPK inhibitor (Compound C); MHY: mTOR agonist (MHY1485). The band density was quantified and expressed as the relative gray value compared with the control.

After intervention, the expression of the autophagy marker protein LC3-II in MHCC-97L cells was detected by Western blot (Figure 5 a). Compared with that in the control group, the LC3-II expression in MHCC-97L cells was significantly increased after treatment with radiation or SSd alone. In the radiation and SSd combined group, LC3-II expression was further increased. However, after the addition of siRNA-P53, MHY1485 or Compound C, LC3-II expression was decreased.

mTOR is a key molecule regulating cell autophagy. To investigate the mechanism of how SSd increases radiosensitivity by inducing autophagy in hepatoma cells, we detected changes of mTOR expression and its downstream proteins by Western blot. mTOR expression was not significantly changed in MHCC-97L hepatoma cells treated with radiation or SSd alone, but p-mTOR expression was lower than that in the control group; in the radiation and SSd co-treatment group, p-mTOR expression was further reduced. Further studies have found that the addition of siRNA-P53 or Compound C can increase p-mTOR expression in MHCC-97L hepatoma cells, as described in the literature 14. In addition, after the combined treatment with radiation and SSd, the expression of p62, a downstream protein of mTOR, was decreased, and the expression of Beclin-1 was increased. Meanwhile, Apoptosis-related protein of cleaved caspase-3 and cleaved PARP expression were significantly higher in the combined group than in the control group. However, after the addition of the MHY1485, siRNA-P53 or Compound C, with the increase of p-mTOR expression, the inhibitory effects of SSd on the above proteins in hepatoma cells were partially reversed (Figure 5). Therefore, SSd increases radiation-induced apoptosis of hepatoma cells, which may be related to promoting autophagy via inhibiting mTOR phosphorylation.

## Discussion

Autophagy is a protein degradation pathway used by eukaryotic cells to remove intracellular damage, thereby maintaining homeostasis. This process plays an important role in the physiology and pathology of cells, especially in the development of malignant tumors 20. However, excessive autophagy upregulation can also cause autophagic cell death, also known as type II programmed cell death, which is a programmed cell death pathway different from apoptosis 21.

Radiation resistance of tumors is an area of focus for oncologists. The level of autophagy is significantly increased during radiotherapy, and normal induction of autophagy can result in a better therapeutic effect. After radiation, the ability of tumor cells to self-renew is destroyed; in addition, cell mitosis is blocked, and autophagy beyond what can be reversed by the cells leads to tumor cell death. Wu 22 explored the relationship between radiation and autophagy in oral squamous cell carcinoma cell lines (OC3), and the results showed that the survival rate of OC3 cells was further reduced after co-treatment with radiation and the autophagy inducer rapamycin, suggesting that radiation and autophagy induction have a synergistic effect on OC3 tumor cell growth. Our results showed that the clone formation of both SMMC-7721 and MHCC-97L hepatoma cells after radiation exposure was significantly inhibited, and the inhibition of hepatoma cell clone formation was further increased after the addition of SSd. However, after the addition of the autophagy inhibitor chloroquine, the inhibitory effect of SSd combined with radiotherapy on hepatoma cell proliferation was partially reversed. In addition, we found that the promoting effect of SSd on apoptosis in hepatoma cells induced by radiotherapy was significantly reversed after the addition of chloroquine or the mTOR agonist. Therefore, the results of the study indicate that the inhibition of autophagy reduces the sensitization effect of SSd on hepatoma cell radiotherapy.

The observation of the formation of autophagosomes by electron microscopy revealed that the number of autophagosomes in hepatoma cells increased after radiation, and the number of autophagosomes further increased after the addition of SSd. These results indicate that SSd can enhance the autophagic formation of radiation-induced liver cancer cells. Based on the transmission electron microscopy results, we further quantified the changes of autophagy by laser confocal microscopy. The results showed that SSd can promote radiotherapy-induced autophagy in hepatocarcinoma cells,which was consistent with the electron microscopy results. However, the promoting effect of SSd on radiation-induced autophagy in hepatoma cells can be reversed by the mTOR agonist, Compound C or p53 interference. These results suggest that mTOR may be a key target for SSd-induced autophagy in hepatoma cells and enhance radiation sensitivity.

The process of autophagy is regulated by a variety of signaling pathways and autophagy-related genes, including continuous processes such as initiation, elongation, maturation, and degradation. The activation of autophagy is dependent mainly on the mTOR signaling pathway, in which mTORC1 (mTOR complex 1) plays an important role 23. Our results showed that the expression of mTOR was not significantly changed in MHCC-97L hepatoma cells treated with radiation or SSd alone; however, the expression of p-mTOR was lower than that in the control group, especially in the radiation and SSd co-treatment group. In addition, after the intervention of MHY1485, Compound C or siRNA-P53, the inhibitory effect of SSd and radiotherapy co-treatment on p-mTOR expression in hepatocellular carcinoma cells was decreased, indicating that the intervention of SSd and radiotherapy on mTOR expression occurs at the phosphorylation level.

The autophagosome extension phase is achieved mainly by a ubiquitination complex system, which includes the ATG12 binding process and the LC3 modification process. LC3 is present mainly in the cytoplasm in the form of LC3-I. During autophagy, cytosolic LC3 (LC3-I) is transformed into membrane-bound LC3 (LC3II), which is then located in the autophagosome membrane and outer membrane. Therefore, the detection of LC3-II expression levels by immunoblotting can reflect the number of autophagosomes 24, 25. Our results showed that the expression of LC3-II in MHCC-97L cells was significantly increased in the radiotherapy group after the addition of SSd. However, after the addition of the MHY1485, Compound C or p53 interference, the expression of LC3-II was reduced. Therefore, SSd promotes radiation-induced autophagy in hepatoma cells, and increased expression of LC3-II can be reversed by mTOR agonists.

LC3-II also binds to p62/sequestosome-1 (SQSTM1), and p62 not only transports proteins to the proteasome but also promotes the autophagic degradation of ubiquitinated proteins. When autophagy is normal, p62/SQSTM1 can be degraded, but when autophagy is absent or impaired, p62 will accumulate 26, 27. Our results showed that the expression of p62 was decreased, and the expression of Beclin-1 was increased after MHCC-97L hepatoma cells were treated with the combination of radiation and SSd. Meanwhile, apoptosis-related protein of cleaved caspase-3 and cleaved PARP expression were significantly higher in the combined group than in the control group. However, after the addition of the MHY1485, Compound C or p53 interference, the effects of SSd on the inhibition of p62 expression and the enhancement of Beclin-1, cleaved caspase-3 and cleaved PARP expression were partially reversed.

Our results preliminarily showed that SSd can increase the radiosensitivity of hepatoma cells, which is related to the inhibition of mTOR phosphorylation by SSd and the promotion of autophagy in hepatoma cells. However, the occurrence and development of liver cancer is a multi-gene, multi-target complex process involving multiple signaling pathways. The current data are only the results of our preliminary research. In future studies, we will further investigate the relationship between SSd-induced autophagy and apoptosis *in vivo*, and their role in increasing radiosensitivity of hepatoma cells.
